# Exploring kitten socialisation practices and welfare implications within a Swedish breeding association

**DOI:** 10.1017/awf.2026.10062

**Published:** 2026-01-22

**Authors:** Elin Netti Hirsch, Helena Sunning, Maria Andersson

**Affiliations:** Department of Applied Animal Science and Welfare, Swedish University of Agricultural Sciences, Skara, Sweden

**Keywords:** Animal welfare, attitudes, breeder, domestic cat, early experiences, knowledge

## Abstract

A kitten’s early experiences have lasting effects on adult behaviour and welfare. Domestic cat (*Felis silvestris catus*) breeders play an important role in shaping these outcomes through their knowledge and understanding of early environment and socialisation. This study aimed to investigate Swedish pedigree cat breeders’ knowledge, attitudes and current socialisation practices. An online survey was distributed via social media to active Swedish breeders of pedigree cats (n = 133). The data were analysed primarily using descriptive statistics with several areas for improvement being identified. More breeders stated the importance of multiple people handling the kittens than they applied in practice. Significantly more breeders reported that it was important for kittens to interact with other companion animals than they demonstrated in practice. Gaps in knowledge were identified regarding the optimal age for socialisation, handling quantity, and the heritability of traits relating to the approach to new experiences. Breeders generally expressed positive attitudes towards socialisation and wished they could have prioritised it more. These findings have important implications for animal welfare, as insufficient early socialisation can increase the risk of fear-related behaviours and reduced overall cat welfare later in life. In conclusion, this study, although based on a convenience sample, provides an important first step towards understanding and improving kitten socialisation practices in Sweden and elsewhere.

## Introduction

Undesired behaviours, stress and fear are increasingly reported by cat guardians (Levine 2008; Sandøe *et al.* 2017). These issues may stem from a true increase in prevalence, changes in husbandry practices, or greater awareness of such problems. Fear, anxiety and stress appear broadly similar both in terms of mechanisms and experience. Still, stress is mostly framed within the context of responses that help an animal to maintain homeostasis (McMillan 2005) via activation of emotions such as fear (Levine 2008). Besides welfare issues with regards to experiences of fear, undesired behaviours, often shaped by early life experiences, may lead to cats being rehomed, relinquished to shelters, or euthanised (Heath 2007; Amat *et al.* 2009). Rehoming and being removed from a familiar environment will include stressors that can negatively impact cat welfare (Stella & Croney 2019). The shelter environment includes several factors known to negatively influence cat welfare, such as the presence of unknown cats (Kessler & Turner 1999; Ottway & Hawkins 2003), changes in handling and routine (Carlsted *et al.* 1993; Stella *et al.* 2011) and competition for space and resources (Gourkow & Fraser 2006). Early life experiences will have long-lasting effects on behaviour (Karsh & Turner 1988), environmental adaptability (Feuerstein & Terkel 2008; Bradshaw *et al.* 2012), mood regulation, cognitive abilities and social relationships (Sanchez 2006). Early experiences shape an individual’s ability to cope with challenges later in life (e.g. McCune 1995; Casey & Bradshaw 2008), like stress and fear, which otherwise can lead to undesired behaviours. A risk that can be reduced by preparing and familiarising cats with stimuli they will encounter later in life through appropriate socialisation.

For domestic cats (*Felis silvestris catus*), the primary socialisation period towards humans has been determined as taking place between 2 and 7 weeks (Karsh & Turner 1988). Handling during this period is required for cats to become comfortable with humans in general and has a clear effect on whether a cat will actively seek out human contact (McCune 1995). Several factors have been shown to influence successful socialisation, including the presence of a calm mother (Crowell-Davis 2007), the temperament of the father (Turner *et al.* 1986; McCune 1995), the quality of handling (such as duration [McCune *et al.* 1995], number of handlers [Collard 1967]), exposure to different rearing environments (Feuerstein & Terkel 2008; Amat *et al.* 2009) and weaning age (Ahola *et al.* 2017). For instance, early weaning (prior to 8 weeks) is linked to undesired behaviours such as human-directed aggression as well as stereotypic behaviour (Ahola *et al.* 2017). Kittens raised without the mother, whether as a singleton or with sibling(s), displayed signs of distress more quickly, such as struggling when handled and vocalised more frequently during separation than kittens raised together with their mother (Martínez-Byer *et al.* 2023). Kitten socialisation is therefore vital for proper development and successful future interactions with humans (Karsh & Turner 1988). Similarly, it is important during this period for the kitten to undergo positive experiences regarding scenarios it may encounter in later life, such as children, visits to the veterinarian and encountering other species. In kittens, only individuals with early social experience of puppies interacted affiliative with dogs as cats do not seem to generalise their intraspecific social experiences to interspecific interactions (Fox 1969). In general, situations that kittens are positively exposed to during the socialisation period are handled better later in life, with fewer signs of fear and stress (Turner & Bateson 2000). On the other hand, inadequate socialisation is associated with increased fearfulness (Casey & Bradshaw 2008). Without proper preparation for a life with humans, i.e. positive experiences, cats are at risk of experiencing fear and stress, and consequently decreased welfare throughout life. Less is known regarding proper intraspecific socialisation, but through, e.g. social play between 8–16 weeks of age (West 1974), kittens are less prone to inappropriate social behaviours, such as aggression, and will get along with other cats better (Crowell-Davis *et al.* 2004; Ramos 2019).

Inadequate socialisation increases the risk of fear and undesired behaviours (Casey & Bradshaw 2008) and the risk of being relinquished, rehomed or euthanased (O’Handley *et al.* 2021). Well-socialised cats show reduced risks of the above and tend to be adopted quickly if relinquished (Brown & Stephan 2021).

There are no studies examining the long-term effects of differences in socialisation experiences in terms of cat behaviour (Mendl & Harcourt 2000) or the cat-human relationship (Casey & Bradshaw 2008). In general, the long-term effects of specific early experiences in animals are not well understood (Stamps & Groothuis 2010). However, the relationship with humans appears to continue developing until kittens are four months of age (Lowe & Bradshaw 2002). In Sweden, kittens’ entire primary socialisation period takes place at the breeder. Legally (in typical cases) kittens must remain with their mother and siblings until 12 weeks of age and recommendations for breeders belonging to Fédération Internationale Féline (FIFe) is for weaning to occur no earlier than 14 weeks. Given that this period takes place entirely under the care of the breeder, their knowledge of and attitudes towards socialisation and early experiences should not be overlooked. Yet, despite this, remarkably little is known regarding breeders’ understanding of such factors or their practical implications. A breeder’s influence on a cat’s future behaviour and relationship with humans cannot be underestimated.

The purpose of this study was to describe how Swedish pedigree cat breeders care for their kittens during the socialisation period, with a focus on factors pertinent to socialisation, as well as the breeders’ knowledge, attitudes, and perspectives on the subject. As part of a wider investigation into cat socialisation, the long-term goal of which being increased knowledge regarding how a cat’s early experiences impact stress, fear and undesired behaviours later in life, the results will contribute to knowledge related to the welfare of domestic cats. The following research questions were formulated: ‘What early experiences are provided for kittens from Swedish pedigree cat breeders?’; ‘What knowledge do Swedish pedigree cat breeders have about the socialisation period?’; and ‘What attitudes do Swedish pedigree cat breeders have towards kitten socialisation and how is socialisation prioritised?’

## Materials and methods

### Ethical considerations

No ethical approval was needed for our study since it was based on an online survey that conformed to the standard guidelines at the Swedish University of Agricultural Sciences and followed GDPR. The survey did not collect any sensitive personal information, and participants were informed about the purpose of the study and provided informed consent for the collection and processing of data prior to participation. Although the study did not involve direct interaction with animals, the findings have implications for improving animal welfare through better early-life practices. Care was taken to present the findings constructively, with an emphasis on supporting breeders in enhancing animal welfare.

### Survey design and data collection

An online survey was developed and administered using Netigate (Netigate AB, Stockholm, Sweden) and can be seen in the Supplementary material. The survey was open from October 1st to October 11th, 2021, and, to compensate for this relatively short data collection period, we underwent collaboration with The National Association of Swedish Cat Clubs (SVERAK) which provided direct access to registered cat breeders via their established communication channels. This enabled efficient dissemination of the survey to our target population. Reminders were sent directly to breed clubs using social media one week and one day before the survey’s closing date to help increase response rates. Questions were designed using guidelines for questionnaire construction from Statistics Sweden and based on a literature review. Multiple-choice questions, with fixed response options, were primarily used, but some free-text questions were also included, as well as one question with a ranking scale.

The survey was divided into three main sections: basic information; breeding practices; and knowledge regarding kitten socialisation. The first section aimed to describe the respondents’ breeding activities (demographic data, cat breed, number of cats, etc). After respondents specified the cat breed they primarily bred, they were asked to answer the remaining survey questions based on that specific breed. The second section related to present-day breeding practices, with a focus on aspects related to kittens (amount of handling, contact with children, contact with other animal species, etc). The choice of items related to socialisation practices included in the survey was selected based upon having previously been related to socialisation outcome (e.g. number of handlers [Collard 1967] and handling time [McCune *et al.* 1995]) or relating to common husbandry practices in Sweden, e.g. around 20% of households with children also have cats (SCB 2012). This aimed to determine what early experiences kittens received from breeders. The third section related to knowledge about kitten socialisation and included factual questions intended to gauge breeders’ knowledge regarding socialisation. Several questions scattered throughout the different categories aimed to assess breeders’ attitudes toward socialisation.

Response options, placed in no particular order, were randomly organised per respondent to avoid position effects. Response options with numerical scales were provided in ascending order and questions with yes or no response options were consistently presented for all respondents. The response options ‘do not know’ or ‘other’ were available for all questions, which allowed for inclusion of open-text answers. The responses relating to handling time were later categorised into four categories based upon earlier research examining its influence on socialisation (Karsh & Turner 1988). These categories were less than recommended (< 40 min per day), recommended (40–60 min per day), more than recommended (> 60 min per day) and other.

A pilot test was conducted prior to delivering the actual surveys. The survey’s linguistic content was tested by sixth-year veterinary students (n = 4) who owned one or more cats with content accessibility for the target group tested by one SVERAK board member. The survey was revised whereby a question regarding weaning was changed from multiple-choice to free-text and the language modified for the target group (i.e. concepts were explained).

Eligibility criteria included being an adult, an active breeder of pedigree cats, based in Sweden and a member of SVERAK who engages in earlier breeding of pedigree cats. In 2020, there were 1,648 breeders with registered cattery names and 137 breeders without registered cattery names in SVERAK, resulting in a total target group of 1,785 breeders (C Alsmark, personal communication 2022). According to statistics from the mandatory Swedish cat registry (administered by the Swedish board of Agriculture), pedigree cats constituted 23% of registered cats in 2024 (Swedish Board of Agriculture 2025). Currently, there are no data regarding the number of non-pedigree cat breeders in Sweden.

Breeders were recruited using a non-probability convenience sampling method through distributing the survey link mainly via social media (Facebook) connected to SVERAK and cat clubs as well as breed rings affiliated with SVERAK. Further distribution through snowball sampling (Johnson 2014) likely occurred. The survey was also promoted by distributing flyers at the “Sydkatten” cat exhibition in Malmö, Sweden, on October 2nd and 3rd, 2021. Facebook reminders were posted both one week and one day prior to the survey closing date.

Results were collated in Microsoft Excel® 2010. Free-text answers from the ‘other’ or ‘do not know’ categories were recorded into existing options, post-survey, when synonymous or clear that they belonged to an available option. These answers are included and reported in the results in the corresponding categories. Chi-squared tests (α = 0.05) were performed in Minitab® (version 2020) to examine whether respondents’ attitudes toward socialisation aligned with their actual socialisation practices.

## Results

In total, 187 cat breeders started the questionnaire. The results are based on 133 fully completed surveys. The average response time was approximately 15 min.

### Breeder demographics and practices

Responding breeders (Table 1) represented all counties in Sweden besides two, with the majority belonging to the three largest counties: Stockholm County (n = 28; 21%); Västra Götaland County (n = 23; 17%); and Skåne County (n = 12; 9%).

**Table 1.** Pedigree cat breeder (n = 133) demographics, experience and factors related to the kittens’ early environment. Where the number of respondents differs from the total sample due to follow-up questions or non-applicability, the number of respondents is shown in parentheses

Variable	Number of breeders	Percentage
For how long have you been breeding pedigree cats?		
< 1 year	5	4
1–3 years	25	18
4–6 years	29	22
7–10 years	18	14
> 10 years	56	42
How many litters have you bred in total?^1^		
1–3 litters	27	20
4–6 litters	27	20
7–10 litters	27	20
11–15 litters	16	12
16–20 litters	5	4
> 20 litters	29	22
How many litters do you normally bred per year?		
< 1 litter	25	19
1–2 litters	84	63
3–4 litters	19	14
5–6 litters	1	1
> 6 litters	4	3
In what environment is your breeding situated?		
City	35	26
Neighbourhood of houses/townhouse	53	40
Rural	45	34
Do your cats have access to the home?		
Yes	131	99
No^2^	2	1
Are their children living in the household?		
Yes	49	37
No	84	63
If yes, what age group(s) (n = 49)		
only younger (0–6 years)	9	19
only older (7–18 years)	32	65
both (0–18 years)	8	16
Do the kittens come into regular contact with children?^3^		
Yes	79	
No	40	
If yes, what age group(s) (n = 79)		
only younger (0–6 years)	14	18
only older (7–18 years)	36	45
both (0–18 years)	29	37
Do you keep other animals besides cats?		
Yes	50	38
No	83	62
If yes, what animal species? (n = 50)		
Dogs only	28	56
Dogs and other	9	18
Other (not dogs)	13	26
Do your kittens come into regular contact with other companion animals?^4^		
Yes	45	34
No	86	65

1 Two respondents excluded here due to free-text answers that did not align with question format.

2 One respondent stating that females had full access to home, while males lived in a separate space.

3 14 respondents excluded here due to free-text answers that did not align with the question.

4 Two respondents excluded due to free-text answers that did not align with question format.

All respondents housed at least one cat at the time of the survey. This excludes cats kept in foster families and kittens intended for sale. The most frequently occurring category was keeping 4–6 cats (n = 56; 42.1%), followed by 2–3 cats (n = 41; 30.8%) and 7–9 cats (n = 26; 19.5%). More respondents kept ten or more cats (n = 9; 6.8%) compared to only one cat (n = 1; 0.8%). Respondents represented 16 different cat breeds approved by SVERAK with the most common breed being the Siberian cat (n = 17; 12.8%), followed by the Sacred Birman (n = 13; 9.8%), the Devon Rex (n = 12; 9.0%) and the Norwegian Forest cat (n = 12; 9.0%). The remaining 12 breeds were bred by ten respondents or fewer.

When selecting the sire, temperament was given a very high priority by 49 (37%) respondents or a high priority by 51 (38%). Quite high was selected by 19% of respondents and seven (6%) prioritised the sire’s temperament as quite low or very low. The temperament of the queen was prioritised as very high by 63 (47%) respondents or high by 53 (40%) respondents. One respondent prioritised the queen’s temperament as very low. Of the respondents, 90 (68%) believe that sire temperament influences a kitten’s reaction to novel situations, whereas 120 (90%) believe that the queen’s temperament influences a kitten’s reaction to novel situations.

### Breeding practices

Respondents were asked to specify during which period in a kitten’s life they considered socialisation to be most important. The question was presented with an open-ended response option where respondents were encouraged to provide their own answer. Respondents’ answers varied. The majority provided age intervals in their responses (n = 79; 59.4%), with ages ranging from 0–26 weeks mentioned in various combinations. Among responses containing age intervals, the most common response was 2–8 weeks (8.9%), followed by 2–12 weeks (7.6%), 3–12 weeks (7.6%), and 4–12 weeks (7.6%). Among responses that included age intervals, intervals starting at two weeks of age were the most common (32.9%). Similarly, twelve weeks of age was the upper age limit most provided by respondents (32.9%). Of all respondents (n = 133), three specified the scientifically supported socialisation period between 2–7 weeks of age. However, 20.3% of the responses included this period.

The responses that did not contain age intervals were categorised as ‘other’ (40.6%). Among respondents in the ‘other’ category, the majority provided only a single number (72.2%), making these responses difficult to interpret based on the question. Of the remaining ‘other’ responses, eight respondents stated that kittens should be socialised from birth and seven respondents beginning at an age between 2–8 weeks. Neither group specified an upper limit.

The majority (85.7%) of respondents began handling their kittens at birth. A few stated that they started handling the kittens regularly at one week (4.5%), two weeks (5.3%), or three weeks of age (3.8%). No respondent began handling their kittens later than three weeks. One ‘other’ response could not be interpreted based on the question. Handling was defined in the survey as when the kitten is in physical contact with a human, such as being handled, carried, sitting on a lap, or being petted. Daily weighing was included in the concept for this specific question.

Significantly more breeders reported that they considered (n = 124; 93.2%) it important for kittens to be handled by several people, than provided (n = 102; 76.7%) handling by several people during the socialisation period (χ^2^ [1, n = 261] = 22.883; *P* < 0.001). Close to a quarter (n = 31; 23.3%) of respondents stated that kittens were handled primarily by one person. Four respondents replied that kittens should be handled by the same person during the socialisation period and two respondents replied that the number of handlers did not matter. One respondent selected ‘do not know’ and two respondents’ answers could not be categorised.

Regarding the amount of time, per day, respondents spent handling their kittens, only an open-ended response option was provided in which respondents were encouraged to provide their own answers. Forty-eight (36.1%) handled their kittens for < 40 min per day, 29 (21.8%) did so 40–60 min per day and 27 (20.3%) respondents > 60 min per day. Twenty-nine (21.8%) answers encompassed more than one time category. One respondent’s answer could not be categorised. When asked how many minutes per day a kitten should be handled during the socialisation period, 41 (30.8%) respondents stated < 40 min per day, 28 (21.1%) respondents 40–60 min per day and 33 (24.8%) respondents > 60 min per day. Three respondents responded that they ‘do not know’. Seven answers encompassed more than one time category and 21 (16%) could not be categorised.

All kittens in a litter were handled for approximately the same time by 97 (72.9%) respondents. Twenty-two (16.5%) respondents did not handle all the kittens for the same amount of time. Amongst these, six mentioned that shy/careful kittens were actively handled more, or that it depended upon the individual (n = 4) and cases where the kitten needed additional feeding (n = 6). Five respondents stated, ‘do not know’ and nine ‘other’ respondents’ answers could not be categorised.

Significantly more (71%) respondents stated that it was important for kittens to encounter other companion animals besides cats, compared to those actually providing this (32%) opportunity (χ^2^ [1, n = 267] = 39.75; *P* < 0.001). None of the respondents considered it to be bad. However, 28 (21.1%) respondents stated, ‘do not know’. Nine respondents provided ‘other’ answers, whereby two mentioned that, from a social perspective, it was positive for kittens to encounter other animals, but it was negative from a disease control perspective. The remaining seven ‘other’ respondents’ answers could not be categorised.

The majority of respondents (n = 125; 94%) attended a veterinarian for vaccination, ID and examination. Five respondents had the veterinarian come to the breeder and two performed vaccinations and ID themselves but took the kittens to a clinic for examination. One respondent answered that protocols differed depending on pricing quoted.

Eighty-eight respondents (79%) weaned (separated the kittens from the queen) between the ages of 12 and 14 weeks while 17 (12.8%) kittens were weaned later than 14 weeks. Of respondents, 13 answered that they weaned kittens prior to 12 weeks of age. As the question was free-text, nine respondents provided answers showing the age interval and six provided answers not containing any age.

### Attitudes

In ranking five aspects related to breeding from highest to lowest priority, finding a good home and socialisation, were ranked highly by most respondents (Table 2). When asked if a respondent would have liked to prioritise socialisation more, 70 (53%) respondents answered ‘no’, 56 (42%) ‘yes’ and 7 ‘I do not know’.

**Table 2.** Swedish breeders’ (n = 133) ranking of five different aspects relating to cat breeding. Bold indicates the ranking alternative selected by most respondents for each aspect. Respondents had to prioritise all aspects according to one rank each

Highest (1) to lowest (5) priority	1	2	3	4	5
Finding a good home/owner	**57 (42.8%)**	35 (26.3%)	36 (27.1%)	4 (3%)	1 (0.8%)
Socialisation	41 (30.8%)	**69 (51.8%)**	21 (15.8%)	1 (0.8%)	1 (0.8%)
Disease control and hygiene	32 (24.1%)	24 (18%)	**62 (46.6%)**	11 (8.3%)	4 (3%)
Coat colour and markings	1 (0.8%)	4 (3%)	6 (4.5%)	59 (44.4%)	**63 (47.3%)**
Economy (monetary earning)	2 (1.5%)	1 (0.8%)	8 (6%)	58 (43.6%)	**64 (48.1%)**

When asked if there is accessible information in Swedish regarding socialisation of kittens, 50 (37.6%) respondents stated, ‘do not know’, 48 (36.1%) ‘no’ and 29 (21.8%) ‘yes’. Six respondents answered ‘other’. Of the 29 respondents that answered yes, 28 specified their sources of information with the internet being the most common (67.9%), followed by other breeders or breed clubs (60.8%), books and literature (39.3%), SVERAK (21.4%) and courses and lectures (17.9%). Sixty-four respondents (48%) did not know if they believed that Swedish pedigree cat breeders had enough knowledge about socialisation of kittens with 47 (35%) answering that they did not and 22 (17%) responding that yes they did.

## Discussion

The socialisation period of pedigree cats in Sweden was explored via an online survey with early experiences of kittens investigated along with cat breeders’ attitude and knowledge in relation to kitten socialisation. The findings showed that pedigree kittens are provided with several important early experiences and that breeders generally are in possession of a good level of knowledge regarding socialisation of kittens, as well as positive attitudes related to the subject. Socialisation was set as a high priority by many of the responding breeders. Still, certain aspects regarding to the socialisation of kittens in practice left room for improvement and further research.

Responding breeders overall were experienced, both in terms of their number of years spent as active breeders and the number of litters bred. Many breeders had been active for over ten years and bred more than 20 litters in total. The breeders represented the most common pedigree breeds in Sweden. The four most represented breeds (Siberian cat, Sacred Birman, Devon Rex and Norwegian Forest cat) are all found on the top ten list of most popular breeds registered with SVERAK during 2020 (SVERAK 2024).

All but two respondents stated that cats had access to the full house and in one of these cases, only the males were restricted. Providing full access can promote socialisation as kittens are involved in everyday life and will be automatically exposed to stimuli such as vacuum cleaners, kitchen appliances and screens. Socialising kittens in home environments would appear to produce kittens that are less stressed around humans (Karsh & Turner 1988). However, in her studies, Karsh (summarised in Karsh & Turner 1988) could not provide a reason for this positive effect, but it may be as a result of the combination between copious handling and the opportunity for kittens not only to control the interactions occurring in the environment but also undergo greater exposure to (and therefore become more familiar with) humans and human behaviour. In combination with most respondents having at least two cats at home (not including kittens for sale), most kittens were likely provided the opportunity to interact with cats other than the queen and littermates. This is an important factor for intraspecific socialisation and therefore future social behaviour in cats, especially during the period of peak social play when kittens are between 9 and 14 weeks of age (West 1974). Breeding one or two litters a year, as most respondents, could result in greater time availability for socialising each kitten. Still, depending on the number of persons involved in handling and the time spent at home, this is impossible to estimate based upon the present dataset. Early exposure to humans seems to predict the amount of time a cat chooses to spend together with humans (owners) later in life (Vonk 2023), meaning that adequate socialisation is crucial for the human-animal relationship. Also, the age period during which socialisation is performed can influence future social behaviours and the tendency to actively seek human contact in cats. Amongst responding breeders, three (2.3%) stated correctly the scientifically established key socialisation period between 2 and 7 weeks of age, even if 20% (n = 16) overlapped this period. The time-periods stated by respondents as being most important in terms of handling kittens showed a great deal of variation. This can have a detrimental effect on the socialisation of the kittens, not to mention indicating a considerable gap in knowledge. Still, the practical consequence of this is potentially small since most breeders (85.7%) started handling kittens from birth, even if socialisation *per se* was not the primary intention, i.e. no respondent started to handle kittens regularly later than three weeks of age. The relationship with humans, even if primarily established during the key socialisation period, continues to develop until 16 to 17 weeks of age (Lowe & Bradshaw 2002). Continued active socialisation beyond seven weeks is therefore recommended.

More respondents believed it to be important for kittens to be handled by several individuals than provided handling by multiple people themselves. Limited handling could harm the kitten’s future relationships with humans. Collard (1967) found that kittens that were handled by five people, from 5 to 9 weeks of age, showed less escape attempts when handled by an unknown individual compared to those handled by one person. Interestingly, in that study, one-person kittens attempted to escape from a stranger, on average, the same number of times as kittens that had not been handled at all. Kittens handled by one person showed more affection to the known person and played more in general. Still, today, it is likely more beneficial for kittens to not be stressed by unknown individuals, a situation most cats will encounter at some point. As data collection took place during the autumn of 2021, restrictions and recommendations for social interactions due to the COVID-19 pandemic, could have influenced the opportunities to provide interactions with several individuals. No questions were asked regarding potential changes due to the pandemic.

How much time (in minutes) respondents estimate they spend handling each kitten and how much time they believe a kitten *should* be handled daily, followed a similar distribution when categorised based on Karsh and Turner (1988): up to 40 min per day (less than recommended), 40–60 min per day (recommended) and more than 60 min per day (more than recommended). Overall, most respondents handled their kittens for the recommended amount of time or more (42.1%). Approximately one-third of respondents handled kittens less than 40 min per day. No studies, looking at the amount of handling in the home-environment, are known and results based on experimental studies are difficult to extrapolate. It can be assumed that handling less than 40 min per day could be considered insufficient. Roughly one-third of respondents reported less than the recommended daily duration for kitten handling, both in their own practice and their stated beliefs. This suggests that handling time represents a key area where knowledge and practical socialisation could be enhanced.

As friendliness towards people is related not only to the amount of early socialisation but also genetics, e.g. friendliness of their father (McCune 1995), some kittens might require more positive experiences than others to reach the same level of socialisation. Among respondents, the majority handled all kittens in a litter the same amount of time. Of respondents that did not, providing more handling to shy/careful kittens was mentioned.

The aim of socialisation is to provide kittens with positive experiences related to stimuli, and situations, that they are likely to encounter later in life. Experiences that need to be kept withing each kitten’s ability to cope, to not risk over-exposing kittens, which can result in increased fear levels at the time of adoption in foster kittens (Graham *et al.* 2024). In Sweden, approximately one-third (SCB 2016) of households with cats also have children. Having positive early experiences from interacting with children will therefore likely be beneficial for any cat. Most respondents did not have children living in the house but instead tried to make sure that kittens had the opportunity to regularly come into contact with children by inviting children over during the socialisation period. Interactions with other companion animals were deemed important by a majority of respondents. Early contact with, for instance, dogs have a positive influence upon cats’ interactions with dogs as adults (Fox 1969). However, only around one-third of respondents actually provided interactions with other companion animal species. One-fifth of respondents answered that they did not know whether it was beneficial for cats to come into contact with other companion animals during the socialisation period. This can be interpreted as a lack of knowledge among breeders, or a lack of available information. Also, a few respondents answered in free text that interactions with other companion animals are good from a socialisation perspective but negative from the perspective of hygiene and disease control. Trade-offs between socialisation and aspects relating to disease control is likely a common issue for breeders (Heath 2010). Still, according to Heath (2010), an insufficiently socialised kitten will likely have a worse health status than a well-socialised kitten. Insufficient socialisation will lead to more stress, increased susceptibility to infectious disease, as well as a predisposition to certain urinary tract diseases. These cats are also more prone to engage in negative interactions thereby placing them at greater risk of certain transmissible diseases, such as feline immunodeficiency virus (Yamamoto *et al.* 1989). Still, breeders reported prioritising socialisation before hygiene and disease control.

All but a few respondents took the kittens to a veterinary clinic for vaccination, ID tags and examination. Familiarising kittens with car journeys and handling during health examinations and treatments is important since all cats are likely to encounter these at some point. For many cats, transport and visiting the veterinarian entails several stressors (Pratsch *et al.* 2018). Factors such as difficulties in handling or a cat’s fear of carriers and transportation have been suggested as common sources of stress related to veterinary care (Seksel & Dale 2012). In an online survey of cat owners, the clear majority perceived a visit to the veterinarian as stressful for their cat (Karn-Buehler & Kuhne 2022). Cats are less likely to be taken to the veterinarian compared to dogs and the majority (72%) were taken less than once a year (Lue *et al.* 2008). Avoiding the stress and aversion to cats’ behaviour, in connection to visiting the veterinarian are reasons owners choose not to take, or delay taking, their cat to the veterinarian (Volk *et al.* 2011). Owners also considered potential stress when deciding whether to bring the cat in for vaccination (Habacher *et al.* 2010). The major welfare issue represented by a lack of veterinary care, especially preventive care, can cause both short- and long-term suffering. But, besides these welfare concerns, it is a crime, contravening the Swedish Animal Welfare Act (SFS 2018:1192), to not treat sick or injured animals. And, from a practical perspective, it is much more difficult to examine and diagnose a stressed cat than a calm one. The physiological reactions to stress and fear can also make it harder to diagnose certain diseases, such as diabetes mellitus (Greco 2018).

The question regarding weaning seems to have been misinterpreted by certain respondents. From the free-text responses it was clear that some interpreted the question ‘At what age in weeks are your kittens usually weaned (leave the mother)?’ into meaning when a kitten stops nursing. Others might have weaned earlier than the legal limit, it is unfortunately impossible to determine from the present dataset. Still, the majority did not wean kittens until the age of 12–14 weeks or later. Around 12–14 weeks was also the age most respondents stated as the earliest kittens should be weaned. However, fewer respondents believed that kittens should be weaned later than 14 weeks than stated that they themselves wean kittens later than 14 weeks. Delayed weaning, until 14 weeks, decreased the probability of stranger-directed aggression (Ahola *et al.* 2017). Whether or not delayed weaning, beyond 14 weeks, negatively influences a kitten’s ability to bond to their new family members is yet to be properly investigated. When asked whether the temperament of the parental animals influenced a kitten’s response to novel experiences, more respondents believed there to be an impact from the queen’s temperament than from the sire. Previous studies have shown that the temperament of the sire affects kitten behavioural patterns related to what the authors referred to as “friendliness to humans” (Turner *et al.* 1986). McCune (1995) suggested that this influence was related to the trait of “boldness”, which is a tendency to explore and seek out new experiences. Kittens that are bolder naturally create more opportunities for interactions with humans on their own and thereby receive more socialisation. However, this can be compensated by breeders focusing more on shy kittens as mentioned by six respondents. Respondents also prioritised the temperament of the queen to a greater extent than the sire. Information regarding the importance of the sire’s temperament appears to be lacking to some extent and is undoubtedly an area for improvement within Swedish pedigree cat breeders.

Respondents were generally positive regarding the socialisation of kittens and believed it to be an important aspect of breeding. Finding a good home was the only factor given a higher priority by respondents. The majority of respondents prioritised socialisation higher than, for instance, economy, which was only prioritised to some extent by three respondents. That more than one-third of respondents wished to prioritise socialisation more, in general, also shows an interest amongst breeders and an opportunity to increase knowledge and practice in the area. Enhanced socialisation will likely benefit both future cat-human relationships and cat welfare. Well-socialised kittens often remain well-socialised as adults (Lowe & Bradshaw 2002) and research demonstrates a connection between early socialisation and welfare outcomes. As summarised in a model of kitten socialisation, described by Turner (2021), a well-socialised kitten will not only require fewer positive interactions with a human to become friendly and trusting but will also adapt faster to, for instance, a new home. The opposite is true for not socialised cats, or cats lacking positive experiences with humans.

More respondents replied no, or that they did not know, to the question of whether Swedish breeders of pedigree cats have sufficient knowledge regarding socialisation of kittens. This is problematic as talking to other breeders or cat clubs was the second most common source of information regarding socialisation of kittens. But it also means that spreading information to a subset of breeders, or breed clubs, could be an efficient method of disseminating knowledge within Swedish pedigree cat breeders at large. The view amongst the respondents, that a lack of knowledge exists, could be related to the fact that most respondents believe that there not to be sufficient accessible information regarding socialisation of kittens in Swedish. There are recommendations by Welfare in Pet Trade regarding kitten socialisation in the Eurogroup for Animals (2024), but these would appear to have missed their target group.

### Study limitations

The study design is based upon a convenience sample and self-selection bias could have taken place where primarily highly motivated breeders chose to participate. This could have influenced the results to the more positive side. Still, we believe that the results provide a first indication of socialisation practices amongst cat breeders within SVERAK as the survey encompassed a wide geographical distribution of respondents, reaching all but two counties in Sweden. Open-ended questions require greater effort from respondents and increase the risk of respondents dropping off or providing inadequate answers. Despite this potential drawback, the survey included several free-text questions as these are deemed to provide more accurate and truthful answers by not guiding respondents towards predetermined options. And, in some cases, the opportunity to provide a free-text answer can be motivating since the respondent is invited to write their own story. As many respondents provided extensive answers and included comments for several closed-ended questions, we believe that for most respondents, open-ended questions were motivating.

The two questions regarding weaning of kittens were ambiguous, as became evident during respondent feedback at a cat show where the survey was promoted. The survey defined weaning as the age at which kittens were separated from the queen and moved to new homes. However, several respondents interpreted this as the age when kittens were separated from the queen but remained at the breeder. Using the term “separation from the breeder” or “rehoming age” instead of “weaning” might have prevented this misinterpretation.

### Animal welfare implications

Early-life experiences are crucial for lifelong cat welfare. Since the primary socialisation period (2–7 weeks) and the extended window for socialisation toward humans (up to 16–17 weeks) occur while kittens are still with the breeder, the quality and variety of experiences during this time will have long-lasting effects upon cats’ behavioural development and future welfare. The lack in provisioning socialisation related to certain factors, such as daily handling time and social experiences, may limit a breeder’s ability to support kittens’ behavioural development, potentially increasing the risk of fearfulness, stress-related problems and relinquishment in later life. These are all related to future cat welfare and cat-human relationships. Accessible, practical guidelines in Swedish could empower breeders to provide strengthened socialisation, but such guidelines must be grounded in research to avoid promoting ineffective or potentially harmful practices. For instance, excessive exposure, beyond a kitten’s abilities, can instead increase fear levels at the time of adoption (Graham *et al.* 2024). Enhancing breeder education and support systems can serve as a proactive welfare strategy, reducing the risk of behavioural problems that may otherwise lead to reduced quality of life or early relinquishment. While promoting early positive exposure is likely beneficial, current knowledge is insufficient to specify exactly what experiences are most important or when they should be introduced to maximise welfare outcomes.

## Conclusion

This study identified areas where breeder knowledge appears limited. In connection with breeders reporting a lack of available resources in Swedish, we believe that there to be areas in which guidelines can support breeders in extending their socialisation practices. Guidelines addressing identified areas, such as the amount of daily handling, the timing of the primary socialisation period, sire influence on kitten temperament and interspecies contact could support breeders in strengthening their socialisation practices. As kittens stay with the breeder during the primary socialisation period (2–7 weeks) and throughout most of the time known to allow for additional socialisation towards humans (until 16–17 weeks), the breeder’s role in influencing a cat’s future relationships with humans and welfare, cannot be underestimated. These guidelines would require further studies as we still have no details regarding which experiences should be introduced or if the timing of introduction during the socialisation period influences the result.

## Supporting information

10.1017/awf.2026.10062.sm001 Hirsch et al. supplementary material Hirsch et al. supplementary material

## Supplementary material

The supplementary material for this article can be found at http://doi.org/10.1017/awf.2026.10062.
