# Supplementary material for: Exploring kitten socialisation practices and welfare implications within a Swedish breeding association
Source: Anim Welf. 2026 Jan 22;35:e6. doi: 10.1017/awf.2026.10062 (PMC12836311; doi:10.1017/awf.2026.10062)
Supplement: Hirsch et al. supplementary material [file S0962728626100621sup001.pdf]

Supplementary material

Exploring Kitten Socialisation Practices and Welfare Implications within a Swedish Breeding Association.

Survey questions

1. Basic Information:

- In which county is your cattery located? Options: Roll-down list of Sweden's 21 counties.

- For how long have you been breeding pedigreed cats? Options: <1 year, 1-3 years, 4-6 years, 7-10 years, >10 years, I do not know, Other.

- Approximately how many litters have you bred in total? Options: 1, 2, 3, 4-6, 7-10, 11-15, 16-20, >20, I do not know, Other.

- How many adult cats do you currently keep in your home? (*Do not include cats living with foster homes [breeding lease] or kittens for sale*). Options: 0, 1, 2-3, 4-6, 7-9, >10, Other.

- Which cat breed do you mainly breed at the moment? Options: Roll-down list of all breeds approved by SVERAK.

- How many litters per year do you usually breed? Options: <1, 1-2, 3-4, 5-6, >6, I do not know, Other.

- What type of environment is your cattery located in? Options: City, Neighbourhood od houses/townhouse, Rural

- Do your cats have access to your home, or do they live in a separate area? Options: The cats have access to my home, The cats live in a separate area without access to the rest of my home, Other.

- Do you keep other pets besides cats? Option: Yes, No, Other

-If yes, what animal species. (*Multiple answers possible*). Options: Dog, Rabbit, Horse, Other (with free text option).

- Are there children living in your household? (*Multiple answers possible*). Options: Options: 0–3 years, 4–6 years, 7–12 years, 13–18 years, No, Other.

2. Breeding Practices

- Describe in your own words how you view the handling and socialisation of kittens. Option: Free text.

-Rank how you prioritize aspects of breeding practices of kittens. (*1-highest priority-5 lowest priority, each number used once*) Options: Economy, Colour and pattern, Finding good owners, Biosecurity and hygiene, Socialisation

- At what age do you start handling your kittens regularly? Options: At birth, At one week, At two weeks, At three weeks, At four weeks, Older than four weeks, I do not know, Other.

- Approximately how many minutes per day is each kitten handled? (*Provide answer in numbers.)* Options: Free text.

- Are all kittens in the litter handled for approximately the same amount of time? Options: Yes, No, I do not know, Other. Additional free text option available.

- Are your kittens handled by the same person or several different people? Options: Same person, Different people, I do not know, Other. Additional free text option available.

- Do your kittens regularly come into contact with other pets besides cats? (*With "come into contact with" we refer to a kitten having the opportunity to come in physical contact with or seeing another animal at close range.*) Options: Yes, No, I do not know, Other (with free text option available.)

-If yes, what animal species. (*Multiple answers possible*). Options: Dog, Rabbit, Horse, Other (with free text available).

- Do your kittens regularly come into contact with children? (*Multiple answers possible.*) Option: Options: 0–3 years, 4–6 years, 7–12 years, 13–18 years, No, Other (with free text option available.)

- How do you manage vaccination, ID-marking, and veterinary health checks for your kittens? Options: I go to a vet clinic, The vet comes to my breeding facility, Other (with free text option available.)

- At what age (weeks) are your kittens usually weaned (leave their mother)? (*Provide answer in number.*) Options: Free text

- How do you prioritise the father's temperament when selecting a sire relative to other traits? (*With other traits we mean for example colour, markings, size, economy and practical circumstances*.) Options: Very low, Low, Quite low, Quite high, High, Very high.

- How do you prioritise the mother's temperament when selecting a queen relative to other traits? (*With other traits we mean for example colour, markings, size, economy and practical circumstances*.) Options: Very low, Low, Quite low, Quite high, High, Very high.

3. Kitten Socialisation

- During which period (weeks) of the kitten's life do you believe that socialisation is most important? (*State answer based on kitten age in weeks. Use numbers.*) Option: Free text

- Why is socialisation especially important during this period? Option: Free text

- How many minutes per day should a kitten be handled by people during the socialisation period? (*With "handled" we mean that the kitten is in physical contact with a person. For example, carried, in lap, stroked.)* Options: Free text in numbers.

- Should handling during the socialisation period be by the same person or several different people? Options: Same person, Different persons, I do not know, Other

- Is it beneficial or harmful for kittens to regularly meet other animals besides cats? (*With "come into contact with" we refer to a kitten having the opportunity to come in physical contact with or seeing another animal at close range.*) Options: It is good, It is bad, I do not know, Other

- At what age in weeks should a kitten **at the earliest** be weaned (leave its mother)? (*Respond in numbers*.) Option: Free text

- Does the **father's** temperament influence kittens' responses to new experiences? (*Experiences refer to for example situations, humans, animals, objects etc.*) Options: Yes, No, I do not know, Other

- Does the **mother's** temperament influence kittens' responses? (*Experiences refer to for example situations, humans, animals, objects etc.*). Options: Yes, No, I do not know, Other.

- Would you like to prioritise socialisation more than you currently can? Please motivate your answer. Options: Yes, No, I do not know. Additional free text option for motivation.

- In your opinion, is there easily accessible information for Swedish breeders on socialisation of kittens? Options: Yes, No, I do not know, Other. Additional free text option for motivation.

- In your opinion, do Swedish breeders have sufficient knowledge about kitten socialisation? Please motivate your answer. Options: Yes, No, I do not know. Additional free text option for motivation.

- Would you be open to participate in further research about kitten socialisation? Options: No, Yes
